# Relative cooling power modeling of lanthanum manganites using Gaussian process regression

**DOI:** 10.1039/d0ra03031g

**Published:** 2020-06-01

**Authors:** Yun Zhang, Xiaojie Xu

**Affiliations:** North Carolina State University Raleigh NC 27695 USA yzhang43@ncsu.edu xxu6@ncsu.edu

## Abstract

Efficient solid-state refrigeration techniques at room temperature have drawn increasing attention due to their potential for improving energy efficiency of refrigeration, air-conditioning, and temperature-control systems without using harmful gas in conventional gas compression techniques. Recent developments of increased magnetocaloric effects and relative cooling power (RCP) in ferromagnetic lanthanum manganites show promising results of further developments in magnetic refrigeration devices. By incorporating chemical substitutions, oxygen content modifications, and various synthesis methods, these manganites experience lattice distortions from perovskite cubic structures to orthorhombic structures. Lattice distortions, revealed by changes in lattice parameters, have significant influences on adiabatic temperature changes and isothermal magnetic entropy changes, and thus RCP. Empirical results and previous models through thermodynamics and first-principles have shown that changes in lattice parameters correlate with those in RCP, but correlations are merely general tendencies and obviously not universal. In this work, the Gaussian process regression model is developed to find statistical correlations and predict RCP based on lattice parameters among lanthanum manganites. This modeling approach demonstrates a high degree of accuracy and stability, contributing to efficient and low-cost estimations of RCP and understandings of magnetic phase transformations and magnetocaloric effects in lanthanum manganites.

## Abstract 2

Experimental *vs.* predicted relative cooling power of lanthanum manganites.

## Graphical abstract

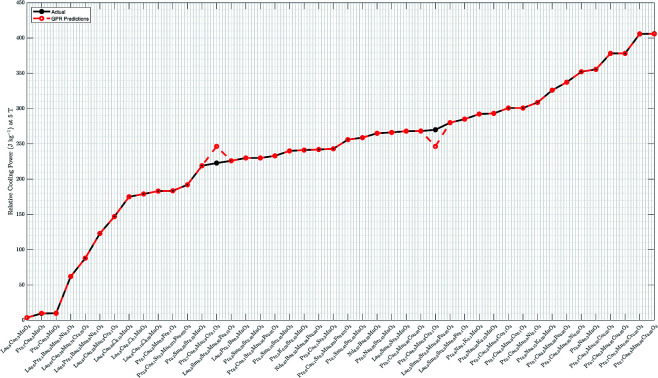

## 1 Introduction

Energy efficiency and sustainability are priority topics in modern society. Refrigeration and air conditioning account for a significant amount of power consumption among various end uses of energy in both commercial and residential areas.^10^ Most refrigeration technology relies on the conventional gas compression (CGC) technique, which has drawn increasing criticisms due to its lack of efficiency and use of air-pollutant gas. Recent developments of magnetic refrigeration (MR) technology, based on the magnetocaloric effect in magnetic materials particularly near room temperature, have offered an exciting alternative to vapor compression refrigeration.^20^ Advantages of MR technology over CGC include, but not limited to, almost ten-fold higher cooling efficiency in magnetic refrigerators, much smaller footprints, complete solid-state operation, and being environmentally friendly.^21^ Furthermore, recent developments in high-temperature superconductors with enhanced critical temperature and magnetic fields that can be generated have prompted developments of high-efficiency MR devices with superconducting magnetic field sources.^8,22,28–30^ An early development of a gadolinium (Gd) rare earth metal with a large magnetocaloric effect (MCE) marked a significant starting point in developing room-temperature MR, but its application in large-scale commercial usage was greatly limited due to the very high price of Gd.^9^ Therefore, numerous research has been conducted to search for new materials with large MCEs, large relative cooling power (RCP), and cheap prices.

Among these materials, ferromagnetic lanthanum manganites, with the general formula, La_1−*x*−*y*_RE_*x*_A_*y*_Mn_1−*z*_TM_*z*_O_3_ where RE is a rare earth element that partially or totally substitutes lanthanum, A is an element of the IA or IIA group, and TM is a transition element that partially substitutes Mn, are of practical importance. These materials have unique properties such as small magnetic and thermal hysteresis, a large MCE around Curie temperature *T*_C_, and a broad working temperature range. Furthermore, manganites are inexpensive to prepare, chemically stable, and highly electrically resistive.^6^ The parent LaMnO_3_ compound is semiconducting and orders antiferromagnetically at 150 K, but a formation of mixed valence in Mn ions *via* a double exchange mechanism between Mn^4+^ and Mn^3+^ can induce ferromagnetism. A wide range of *T*_C_ from ∼150 K to 375 K can be obtained by, for example, substitution of a divalent ion (Ca^2+^, Ba^2+^, Sr^2+^, *etc.*) or a monovalent ion (Na^1+^, K^1+^, *etc.*) for La^3+^, and an excess of oxygen. Furthermore, the ground state of manganites can be tuned by partial substitution of La^3+^ by a trivalent rare earth, or in a La-free Pr or Nd manganites. These perovskite-based structures show lattice distortions as a result of modifications from the cubic structure by the deformation of the MnO_6_ octahedron arising from the Jahn–Teller effect and/or changes in the connective pattern of the MnO_6_ octahedra in the perovskite structure.^26,27^ Values of *T*_C_, magnetic entropy changes Δ*S*_m_, adiabatic temperature changes Δ*T*_ad_, and the resultant RCP are strongly dependent on the doping mechanisms and thus lattice distortions.

Qualitative analysis on the effect of dopant types and levels on RCP of lanthanum manganites has been conducted through experiments, mainly by varying synthesis methods (solid-state reaction, wet chemistry, sol–gel, *etc.*), morphologies (particle size, shape, *etc.*), crystalline states, and final forms (powder, pellet, film, *etc.*).^1,3,4,11–14,16–18,23–25^ Quantitative analysis through thermodynamics models and first-principle models has been utilized to aid the understanding of magnetothermal responses of these materials and facilitate the searching of new candidates for MR devices.^2,5,7,15^ However, these models require a significant amount of data inputs, such as variables for equations of state, exchange coupling energies, and magnetic moments of magnetocaloric materials, which can only be obtained by extensive measurements.

In this work, the Gaussian process regression (GPR) model is developed to elucidate the statistical relationship between RCP and lattice parameters of orthorhombic lanthanum manganites. The model generalizes well in the presence of only a few descriptive features, where intelligent algorithms are able to learn and recognize the patterns. This modeling approach demonstrates a high degree of accuracy and stability, contributing to efficient and low-cost estimations of RCP and understandings of which based on lattice parameters. As one of the computational intelligence techniques, the GPR model has already been utilized in other materials systems to predict significant physical parameters in different fields of applications.^31–44^ On one hand, the model can serve as a guideline for searching for doped-manganites with a large RCP value by screening the lattice parameters. On the other hand, the model can be used as part of machine learning to aid the understanding of the magnetic phase transformation in various types of doped-manganites.

The remaining of this work is organized as follows. Section 2 proposes the GPR model. Section 3 describes the data and computational methodology. Section 4 presents and discusses results, and Section 5 concludes.

## 2 Proposed methodology

### 2.1 Brief description of Gaussian process regression

GPRs are nonparametric kernel-based probabilistic models. Consider a training dataset, {(*x*_*i*_,*y*_*i*_); *i* = 1, 2, …, *n*} where 
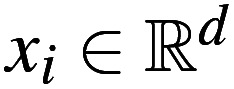
 and 
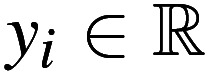
, from an unknown distribution. A trained GPR predicts values of the response variable *y*^new^ given an input matrix *x*^new^.

Recall a linear regression model, *y* = *x*^*T*^*β* + *ε*, where *ε* ∼ *N*(0,*σ*^2^). A GPR aims at explaining *y* by introducing latent variables, *l*(*x*_*i*_) where *i* = 1, 2, …, *n*, from a Gaussian process such that the joint distribution of *l*(*x*_*i*_)'s is Gaussian, and explicit basis functions, *b*. The covariance function of *l*(*x*_*i*_)'s captures the smoothness of *y* and basis functions project *x* into a feature space of dimension *p*.

A GP is defined by the mean and covariance. Let *m*(*x*) = *E*(*l*(*x*)) be the mean function and *k*(*x*,*x*′) = Cov[*l*(*x*),*l*(*x*′)] the covariance function, and consider now the GPR model, *y* = *b*(*x*)^*T*^*β* + *l*(*x*), where *l*(*x*) ∼ GP(0,*k*(*x*,*x*′)) and 
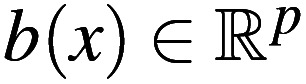
. *k*(*x*,*x*′) is often parameterized by the hyperparameter, *θ*, and thus might be written as *k*(*x*,*x*′|*θ*). In general, different algorithms estimate *β*, *σ*^2^, and *θ* for model training and would allow specifications of *b* and *k*, as well as initial values for parameters.

The current study explores four kernel functions, namely exponential, squared exponential, Matern 5/2, and rational quadratic, whose specifications are listed in eqn (1)–(4), respectively, where *σ*_l_ is the characteristic length scale defining how far apart *x*'s can be for *y*'s to become uncorrelated, *σ*_f_ is the signal standard deviation, 
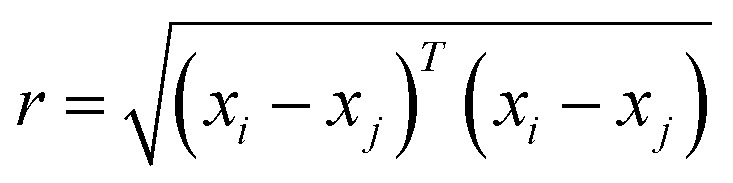
, and *α* is a positive-valued scale-mixture parameter. Note that *σ*_l_ and *σ*_f_ should be positive. This could be enforced through *θ* such that *θ*_1_ = log *σ*_l_ and *θ*_2_ = log *σ*_f_.1
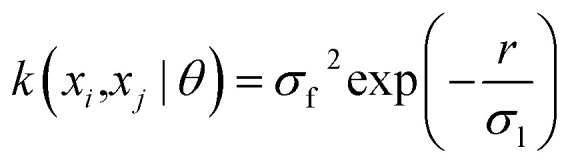
2
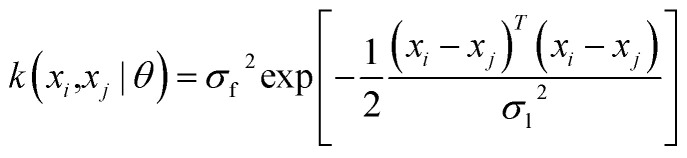
3
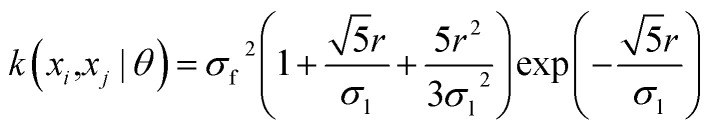
4
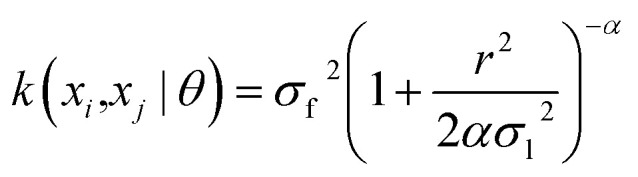


Similarly, three basis functions are investigated here, namely constant, linear, and pure quadratic, whose specifications are listed in eqn (5)–(7), respectively, where *B* = (*b*(*x*_1_), *b*(*x*_2_), …, *b*(*x*_*n*_))^*T*^, *X* = (*x*_1_, *x*_2_, …, *x*_*n*_)^*T*^, and 
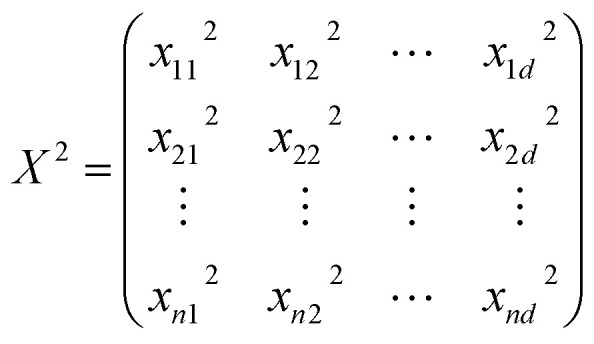
.

5 *B* = *I*_*n*×1_

6 *B* = [1,*X*]

7 *B* = [1,*X*,*X*^2^]

To estimate the GPR model, a Bayesian optimization algorithm is utilized. With a Gaussian process model of *f*(*x*), the algorithm evaluates *y*_*i*_ = *f*(*x*_*i*_) for *N*_s_ points *x*_*i*_ taken at random within the variable bounds, where *N*_s_ points stand for the number of initial evaluation points and 4 is used. If there are evaluation errors, it takes more random points until *N*_s_ successful evaluations are arrived-at. The algorithm then repeats the following two steps: (1) updating the Gaussian process model of *f*(*x*) to obtain a posterior distribution over functions *Q*(*f*|*x*_*i*_,*y*_*i*_ for *i* = 1, … ,*n*); (2) finding the new point *x* that maximizes the acquisition function *a*(*x*). It stops after reaching 30 iterations. The acquisition function, *a*(*x*), evaluates the goodness of a point, *x*, based on the posterior distribution function, *Q*. This work employs the lower-confidence-bound (LCB) acquisition function, which looks at the curve *G* two standard deviations, *σ*_*Q*_, below the posterior mean, *μ*_*Q*_, at each point: *G*(*x*) = *μ*_*Q*_(*x*) − 2*σ*_*Q*_(*x*). Therefore, *G*(*x*) is the 2*σ*_*Q*_ lower confidence envelope of the objective function model. The algorithm then maximizes the negative of *G*: LCB = 2*σ*_*Q*_(*x*) − *μ*_*Q*_(*x*). The optimization is carried out on *σ*, the noise standard deviation. *θ* and *β* are estimated by maximizing the log likelihood function.

### 2.2 Performance evaluation

Performance of the proposed GPR models is evaluated using the root mean square error (RMSE), mean absolute error (MAE), and correlation coefficient (CC) in eqn (8), (9), and (10) respectively, where *n* is the number of data points, *T*^exp^_*i*_ and *T*^est^_*i*_ are the *i*-th (*i* = 1, 2, …, *n*) experimental and estimated magnetic cooling power, and 
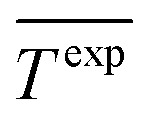
 and 
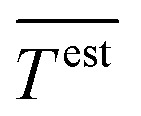
 are their averages.8
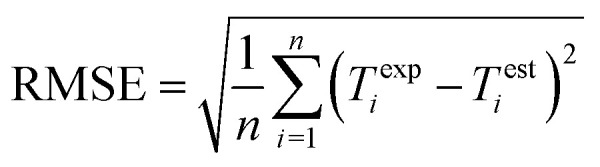
9
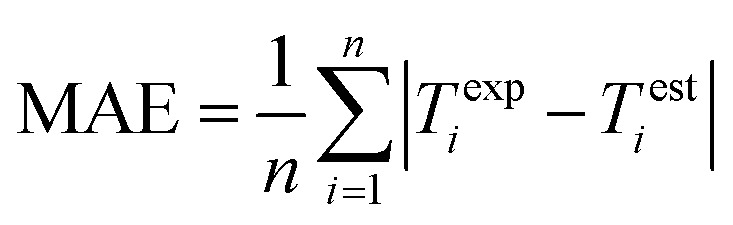
10
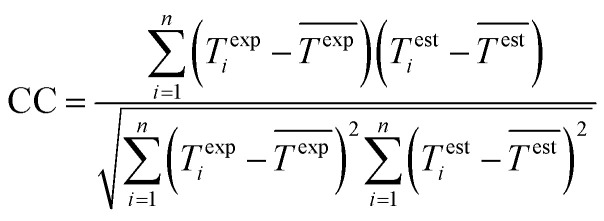


## 3 Empirical study

### 3.1 Description of dataset

**Fig. 1:**
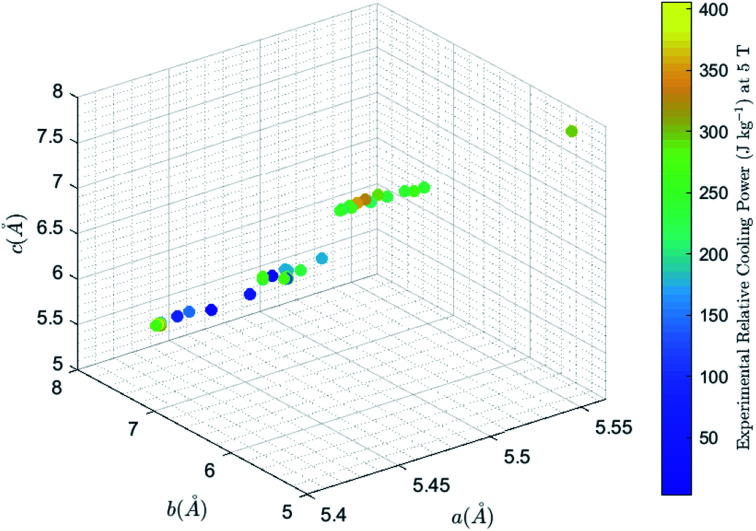
Magnetic cooling power and lattice parameters, *a* (Å), *b* (Å), and *c* (Å).

The experimental data used, shown in Table 1 (Columns 1–5), are obtained from ref. 1, 3, 4, 11–14, 16–18 and 23–25, most of which have been modeled through the support vector machine regression model.^19^ The dataset covers a wide range of doped lanthanum manganites in the form of bulk polycrystalline, single crystal, powders, and sintered pellets, by different synthesis routes including the solid-state reaction, wet-mix processing, and sol–gel processing. RCP values are calculated from Δ*S*_m_ and the full width at half maximum (FWHM) of the Δ*S*_m_*vs. T* curve under Δ(*μ*_0_*H*) of 5 T. Data visualization in Fig. 1 reveals nonlinear relationships, which are modeled through the GPR.

**Table 1:** Experimental data and relative cooling power predictions^a^

Sample	*a* (Å)	*b* (Å)	*c* (Å)	Experimental relative cooling power (J kg^−1^)	Predicted relative cooling power (J kg^−1^)	Reference	Magnetic property
La_0.8_Ca_0.2_MnO_3_	5.4972	7.7771	5.5149	3.67	3.70	13	2nd order FM–PM transition
Pr_0.7_Ca_0.3_MnO_3_	5.4598	7.6741	5.4303	9.72	9.87	25	2nd order FM–PM transition
Pr_0.7_Ca_0.3_MnO_3_	5.4598	7.6741	5.4303	10.00	9.87	4	2nd order FM–PM transition
La_0.6_Pr_0.1_Ba_0.3_Mn_0.7_Ni_0.3_O_3_	5.4813	7.6853	5.4519	62.00	62.02	18	2nd order FM–PM transition
La_0.67_Ca_0.33_Mn_0.75_Cr_0.25_O_3_	5.4419	7.6921	5.4608	88.00	88.02	17	2nd order FM–PM transition
La_0.6_Pr_0.1_Ba_0.3_Mn_0.9_Ni_0.1_O_3_	5.5032	7.7200	5.4690	123.00	123.01	18	2nd order FM–PM transition
La_0.67_Ca_0.33_Mn_0.9_Cr_0.1_O_3_	5.4486	7.7000	5.4673	147.00	147.01	17	2nd order FM–PM transition
La_0.8_Ca_0.05_□_0.15_MnO_3_	5.5253	7.8002	5.5152	175.00	175.01	13	2nd order FM–PM transition
La_0.8_Ca_0.1_□_0.1_MnO_3_	5.5050	7.7866	5.5255	179.00	179.01	13	2nd order FM–PM transition
La_0.8_Ca_0.15_□_0.05_MnO_3_	5.5066	7.7937	5.5084	183.00	183.01	13	2nd order FM–PM transition
Pr_0.7_Ca_0.3_Mn_0.9_Fe_0.1_O_3_	5.4319	7.6753	5.4646	183.50	183.52	23	2nd order FM–PM transition
Pr_0.6_Ca_0.1_Sr_0.3_Mn_0.975_Fe_0.025_O_3_	5.4384	5.4624	7.6776	192.00	192.01	14	2nd order FM–PM transition
Pr_0.2_Sm_0.35_Sr_0.45_MnO_3_	5.4533	5.4380	7.6786	219.03	219.03	16	1st order (AFM, FM)–PM transition
Pr_0.7_Ca_0.3_Mn_0.9_Cr_0.1_O_3_	5.4293	7.6691	5.4552	222.78	246.39	23	2nd order FM–PM transition
La_0.5_Sm_0.2_Sr_0.3_Mn_0.85_Fe_0.15_O_3_	5.5038	7.7388	5.4746	226.00	226.00	1	2nd order FM–PM transition
La_0.6_Pr_0.1_Ba_0.3_MnO_3_	5.5121	7.7508	5.4859	230.00	230.00	18	2nd order FM–PM transition
Pr_0.3_Sm_0.25_Sr_0.45_MnO_3_	5.4623	5.4387	7.6779	230.01	230.01	16	1st order (AFM, FM)–PM transition
Pr_0.6_Ca_0.1_Sr_0.3_Mn_0.95_Fe_0.05_O_3_	5.4382	5.4632	7.6826	233.00	233.00	14	2nd order FM–PM transition
Pr_0.4_Sm_0.15_Sr_0.45_MnO_3_	5.4718	5.4399	7.6773	240.00	240.00	16	1st order (AFM, FM)–PM transition
Pr_0.5_K_0.05_Sr_0.45_MnO_3_	5.4825	5.4429	7.6503	241.20	241.20	12	2nd order FM–PM transition
Nd_0.67_Ba_0.33_Mn_0.98_Fe_0.02_O_3_	5.4917	7.7602	5.5196	242.00	242.00	11	2nd order FM–PM transition
Pr_0.6_Ca_0.1_Sr_0.3_MnO_3_	5.4375	5.4647	7.6814	243.00	243.00	14	2nd order FM–PM transition
Pr_0.6_Ca_0.1_Sr_0.3_Mn_0.925_Fe_0.075_O_3_	5.4427	5.4669	7.6919	256.00	256.00	14	2nd order FM–PM transition
Pr_0.1_Sm_0.45_Sr_0.45_MnO_3_	5.4427	5.4415	7.6800	258.82	258.82	16	1st order (AFM, FM)–PM transition
Nd_0.67_Ba_0.33_MnO_3_	5.4915	7.7591	5.5519	265.00	265.00	11	2nd order FM–PM transition
Pr_0.5_Na_0.05_Sr_0.45_MnO_3_	5.4772	5.4420	7.6441	266.20	266.20	12	2nd order FM–PM transition
La_0.5_Sm_0.2_Sr_0.3_MnO_3_	5.5019	7.7321	5.4696	268.00	268.00	1	2nd order FM–PM transition
Pr_0.7_Ca_0.3_Mn_0.98_Co_0.02_O_3_	5.4303	7.6729	5.4599	268.14	268.14	25	2nd order FM–PM transition
Pr_0.7_Ca_0.3_Mn_0.9_Cr_0.1_O_3_	5.4293	7.6691	5.4552	270.00	246.39	4	1st order FM–PM transition
La_0.5_Sm_0.2_Sr_0.3_Mn_0.95_Fe_0.05_O_3_	5.5036	7.7252	5.4678	280.00	279.99	1	2nd order FM–PM transition
La_0.5_Sm_0.2_Sr_0.3_Mn_0.9_Fe_0.1_O_3_	5.5027	7.7403	5.4740	285.00	284.99	1	2nd order FM–PM transition
Pr_0.8_Na_0.1_K_0.1_MnO_3_	5.4578	5.4588	7.7263	292.24	292.23	3	2nd order FM–PM transition
Pr_0.8_Na_0.05_K_0.15_MnO_3_	5.5641	5.4661	7.7374	293.21	293.20	3	2nd order FM–PM transition
Pr_0.7_Ca_0.3_Mn_0.9_Co_0.1_O_3_	5.4306	7.6705	5.4484	300.80	300.80	23	2nd order FM–PM transition
Pr_0.7_Ca_0.3_Mn_0.9_Co_0.1_O_3_	5.4306	7.6705	5.4484	300.80	300.80	25	2nd order FM–PM transition
Pr_0.7_Ca_0.3_Mn_0.9_Ni_0.1_O_3_	5.4328	7.6892	5.4195	308.70	308.69	23	2nd order FM–PM transition
Pr_0.8_Na_0.15_K_0.05_MnO_3_	5.4507	5.4525	7.7165	325.98	325.97	3	1st order FM–PM transition
Pr_0.7_Ca_0.3_Mn_0.95_Fe_0.05_O_3_	5.4321	7.6743	5.4648	337.40	337.38	24	2nd order FM–PM transition
Pr_0.7_Ca_0.3_Mn_0.95_Ni_0.05_O_3_	5.4295	7.6708	5.4508	352.20	352.19	24	2nd order FM–PM transition
Pr_0.8_Na_0.2_MnO_3_	5.4460	5.4481	7.7113	355.62	355.61	3	1st order (AFM, FM)–PM transition
Pr_0.7_Ca_0.3_Mn_0.95_Co_0.05_O_3_	5.4299	7.6696	5.4572	378.20	378.20	24	2nd order FM–PM transition
Pr_0.7_Ca_0.3_Mn_0.95_Co_0.05_O_3_	5.4314	7.6711	5.4591	378.20	378.18	25	2nd order FM–PM transition
Pr_0.7_Ca_0.3_Mn_0.95_Cr_0.05_O_3_	5.4300	7.6679	5.4572	405.72	405.85	24	2nd order FM–PM transition
Pr_0.7_Ca_0.3_Mn_0.95_Cr_0.05_O_3_	5.4300	7.6679	5.4572	406.00	405.85	4	2nd order FM–PM transition
Mean	5.4636	6.9903	6.1761	239.44	239.44	—	—
Median	5.4520	7.6710	5.4743	249.50	246.39	—	—
Standard deviation	0.0340	1.0638	1.0452	98.66	98.52	—	—
Minimum	5.4293	5.4380	5.4195	3.67	3.70	—	—
Maximum	5.5641	7.8002	7.7374	406.00	405.85	—	—
Correlation coefficient with relative cooling power	−30.47%	−15.67%	14.69%	—	99.87%	—	—

a “*a* (Å),” “*b* (Å),” and “*c* (Å)” are lattice parameters. “Predicted relative cooling power (J kg^−1^)” shows the result from the current work, meaning predicted values based on the Gaussian process regression. “Experimental relative cooling power (J kg^−1^)” and “Predicted relative cooling power (J kg^−1^)” are visualized in Fig. 2. “□” in sample names stands for “vacancy.” “FM” means “ferromagnetism,” “AFM” means “anti-ferromagnetism,” and “PM” means “paramagnetism”.

### 3.2 Computational methodology

MATLAB is utilized for computations and simulations in this work. All observations are used to train the final GPR model given the relative small sample size. The stability of the GPR approach is confirmed by bootstrap analysis.

## 4 Result and discussion

### 4.1 Comparison with previous study

The final GPR model is detailed in Fig. 2, whose performance is compared with that based on the SVM regression in ref. 19.††The comparison is not 100% one-to-one because nine additional observations are used in ref. 19. The data are split into the training and validation sub-samples in ref. 19, where the former has 43 observations and the latter 10. We compare model performance of the GPR with that of the SVM regression^19^ by focusing on their 43 training observations. Switching from the SVM to GPR, the CC increases from 85.07% to 99.87%, the RMSE decreases from 50.5315 to 5.0339, and the MAE decreases from 26.3802 to 1.0923. The GPR model thus provides more accurate relative cooling power predictions than the SVM regression. The result in Fig. 2 shows good alignment between GPR predicted and experimental data.

**Fig. 2:**
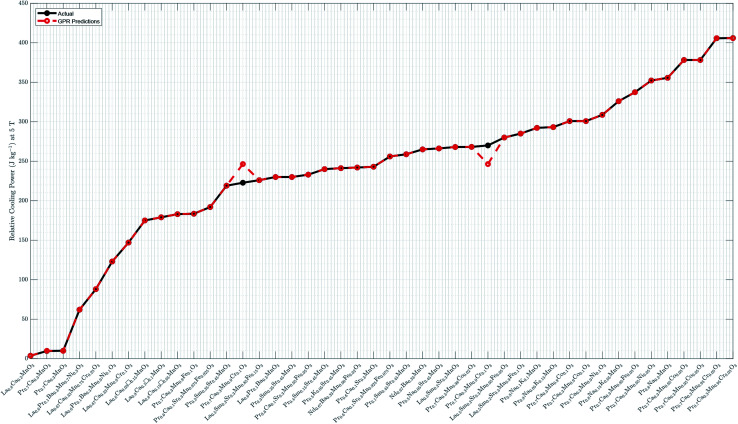
Experimental *vs.* predicted relative cooling power. The GPR model is built using the whole sample with the Matern 5/2 kernel, constant basis function, and standardized lattice parameters. It has a log-likelihood of −813.7988, *β̂* of 233.9604, *σ̂* of 0.9866, *σ̂*_l_ of 0.0053, and *σ̂*_f_ of 89.8778. Detailed numerical predictions are listed in Table 1 (Column 6). “□” in sample names stands for “vacancy”.

### 4.2 Prediction stability

**Fig. 3:**
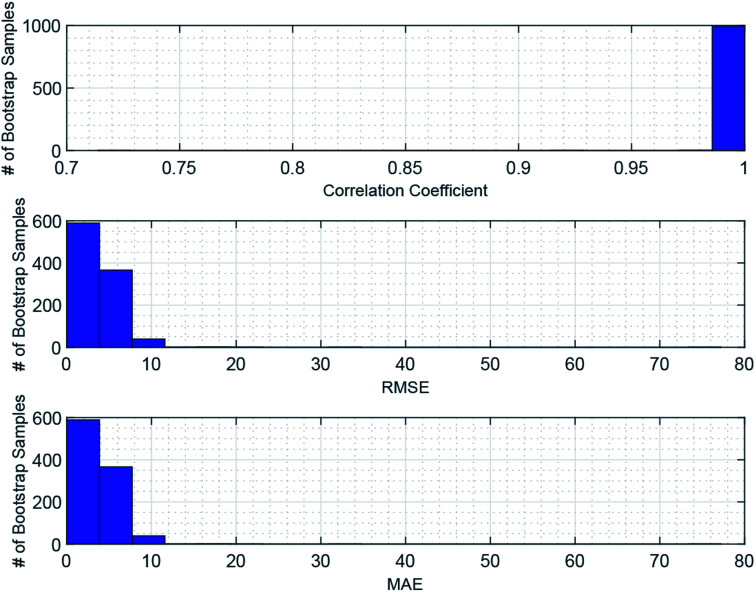
Bootstrap analysis of GPR prediction stability. 1000 bootstrap samples are drawn with replacements from the whole sample. Each bootstrap sample is used to train the GPR based on the Matern 5/2 kernel and constant basis function with lattice parameters standardized, and obtain the associate model performance. The histograms show distributions of the CC, RMSE, and MAE over the 1000 bootstrap samples, whose averages are 99.87%, 2.7915, and 0.9800, respectively.

Given the small sample size (see Table 1) used, the prediction stability of the GPR is assessed through bootstrap analysis in Fig. 3, which shows that the modeling approach maintains high CCs, low RMSEs, and low MAEs over the bootstrap samples. This result suggests that the GPR might be generalized for magnetic cooling power modeling of manganite materials based on larger samples.

### 4.3 Prediction sensitivity

Table 2 shows that GPR predictions are not so sensitive to choices of kernels or basis functions. Because predictions based on different kernel–basis function pairs are so close and nearly visually indistinguishable, they are not plotted for comparisons. However, it is worth noting that estimated model parameters are different across these kernel–basis function pairs.

**Table 2:** GPR prediction sensitivities to kernel and basis function choices^a^

Kernel	Basis function	CC	RMSE	RMSE/sample mean	MAE	MAE/sample mean
Matern 5/2	Constant	99.87%	5.0339	2.10%	1.0923	0.46%
Rational quadratic	Constant	99.87%	5.0458	2.11%	1.3362	0.56%
Squared exponential	Constant	99.87%	5.0345	2.10%	1.1389	0.48%
Exponential	Constant	99.87%	5.1248	2.14%	1.7993	0.75%
Matern52	Linear	99.87%	5.0339	2.10%	1.0947	0.46%
Matern52	Pure quadratic	99.87%	5.0339	2.10%	1.0940	0.46%

a The final GPR model is based on the Matern 5/2 kernel and constant basis function.

## 5 Conclusions

The Gaussian process regression (GPR) model is developed to predict relative cooling power of manganite materials based on lattice parameters. The high correlation coefficient between the predicted and experimental magnetic cooling power, the low prediction root mean square error and mean absolute error, and stable model performance suggest the usefulness of the GPR for modeling and understanding the relationship between lattice parameters and relative cooling power. This modeling approach is straightforward and simple and requires less parameters as compared to thermodynamics models and first-principle models. It can be used as part of computational intelligence approaches for new magnetocaloric materials searches.

## Conflicts of interest

There are no conflicts to declare.

## Supplementary Material
